# A handmade trap for malaria mosquito surveillance by citizens in Rwanda

**DOI:** 10.1371/journal.pone.0266714

**Published:** 2022-05-11

**Authors:** Marilyn M. Murindahabi, Willem Takken, Emmanuel Hakizimana, Arnold J. H. van Vliet, P. Marijn Poortvliet, Leon Mutesa, Constantianus J. M. Koenraadt

**Affiliations:** 1 Laboratory of Entomology, Wageningen University & Research, Wageningen, The Netherlands; 2 College of Sciences and Technology, University of Rwanda, Kigali, Rwanda; 3 Malaria and other Parasitic Diseases Division, Rwanda Biomedical Center, Kigali, Rwanda; 4 Environmental Systems Analysis Group, Wageningen University & Research, Wageningen, The Netherlands; 5 Strategic Communication group, Wageningen University & Research, Wageningen, The Netherlands; 6 College of Medicine and Health Sciences, University of Rwanda, Kigali, Rwanda; Johns Hopkins University, Bloomberg School of Public Health, UNITED STATES

## Abstract

For effective sampling of mosquitoes in malaria surveillance programmes, it is essential to include attractive cues in traps. With the aim of implementing a citizen science project on malaria vectors in rural Rwanda, a handmade plastic bottle trap was designed and tested in the field to determine its effectiveness in capturing adult *Anopheles gambiae* sensu lato, the main malaria vector, and other mosquito species. Carbon dioxide (CO_2_) and light were used as attractive cues. CO_2_ was produced by inoculating sugar with yeast and water. Light was emitted from a torch by light-emitting diodes (LEDs). Under field conditions in rural Rwanda, three handmade trap designs were compared to Centers for Disease Control and Prevention miniature light traps (CDC-LT) in houses. The trap baited with yeast produced CO_2_ and light caught the highest number of mosquitoes compared to the traps baited with light alone or CO_2_ alone. The number of *An*. *gambiae* s.l. in the handmade trap with light and CO_2_ was approximately 9–10% of the number caught with a CDC light trap. This suggests that about 10 volunteers with a handmade trap could capture a similar-sized sample of *An*. *gambiae* as one CDC-LT would collect. Based on these findings, the handmade plastic bottle trap baited with sugar fermenting yeast and light represents an option for inclusion in mosquito surveillance activities in a citizen science context.

## Introduction

Malaria remains a public health concern in many Sub-Saharan African countries including Rwanda. The disease is transmitted by mosquitoes of the genus *Anopheles*, and in Rwanda the most important vectors are *An*. *gambiae s*.*s*. and *An*. *arabiensis* [1]. The country achieved a significant reduction in the burden of malaria through the implementation and scale-up of malaria control interventions from 2005 to 2011 [2]. However, from 2012 to 2016, the country experienced an eight-fold increase in reported malaria cases [3]. This increase in malaria incidence was observed in all 30 districts of the country, thereby putting the entire population at risk of the disease [4, 5].

Integrated vector control currently forms the most effective way to reduce the spread of mosquito-borne diseases such as malaria. These control programmes include mosquito monitoring with the aim to provide information on mosquito abundance and species composition [6]. It thereby enables the assessment of malaria disease risk, and guides vector control in reducing disease transmission and preventing infection [1]. In Rwanda, mosquito monitoring programmes have been established in twelve sentinel sites across the country [3]. However, not all regions of the country benefit from these mosquito monitoring programmes. Factors such as limited funds, limited number of trained entomologists and inaccessibility of some regions hinder the progress towards malaria elimination. In Rwanda, the main methods used for mosquito collection are pyrethrum spray collection (PSC) and human landing catches (HLC) [1, 7–9]. HLC is a collection method based on the use of human volunteers as baits where volunteers collect mosquitoes landing on their exposed legs and feet [10]. As such, the method remains ethically disputed [11], although it remains the most effective estimator of biting intensity currently in use [1, 7–9]. Therefore, other collection methods are desired, but the feasibility, cost and practicability should be considered.

In 2017, the World Health Organization launched the Global Vector Control Response (GVCR) and encouraged countries to employ science and innovation with the aim to bring tangible changes in current vector control programmes [12]. The GVCR sets out the guidance needed to make vector control programmes effective, acceptable and sustainable [6, 12–14]. Among innovative approaches, citizen science can provide benefits in terms of capacity building and tracking mosquito populations, as evidenced by a number of recent publications [15–21]. These initiatives engage volunteer citizens, for example in adult mosquito collections using different trapping techniques [22]. These techniques include the capturing of mosquitoes with hands or with containers against walls [16, 23], or include the submission and identification of adult mosquito pictures or even of mosquito sounds recorded by volunteers [15, 16, 24]. Mosquito traps such as BG sentinel or BG Gravid *Aedes* traps have also been used in a citizen science project to collect *Aedes* species [22].

Interestingly, citizen science approaches for adult mosquito surveillance, and malaria vectors, have hardly been studied in a rural African context. Factors such as cost, ease of use, portability and the effectiveness of the mosquito collection method are crucial when deciding about the sampling approach to employ in a citizen science project, especially in low resource settings [20, 22, 25]. Results of a recent survey based on a participatory approach showed the necessity to provide a simple mosquito sampling tool [25] to capture *Anopheles* mosquitoes in a citizen science context in Rwanda.

Many mosquito sampling tools have been designed on the principle of attraction of mosquitoes towards their hosts. Host-seeking mosquitoes rely on olfaction, visual and thermal cues to locate and identify their vertebrate hosts on which they feed [26–30]. Odours and light as stimuli have been incorporated in many mosquito sampling tools and used to monitor mosquito populations [8, 31]. In addition, visual stimuli such as dark contrast are used by host-seeking mosquitoes to spot a host [29, 30, 32], and thermal sensory information to detect body heat [29]. Carbon dioxide (CO_2_) is one of the main olfactory stimuli involved in the orientation of mosquitoes and other insects that feed blood from their hosts [33]. All vertebrates produce CO_2_ through respiration, and these elevated levels of CO_2_ make mosquitoes more responsive towards volatile host odours. The synergistic combination of CO_2_ and artificial blends of host odours has been extensively studied for deployment in mosquito traps [11, 26, 34–37]. For field sampling purposes, CO_2_ can be produced by fermenting sugar and yeast in water. Hence, sugar-fermenting yeast in a bottle trap has been suggested as a potential cheap and efficient tool for sampling *Anopheles* and other human-biting mosquito species in rural settings [35].

The objective of the present study was to design and evaluate a low cost, easy-to-use mosquito trap to capture adult mosquitoes, including *An*. *gambiae* s.l., for mosquito surveillance in rural Rwanda. The goal was to employ this trap in a larger one-year citizen science programme that was co-developed with the local population [25] and which ran from November 2018 to October 2019. Trap designs were evaluated under field conditions by comparing them to Centers for Disease Control and Prevention miniature light traps (CDC-LT), which are considered the gold standard sampling method [9, 38].

## Materials and methods

### Field experiments

**Fig 1:**
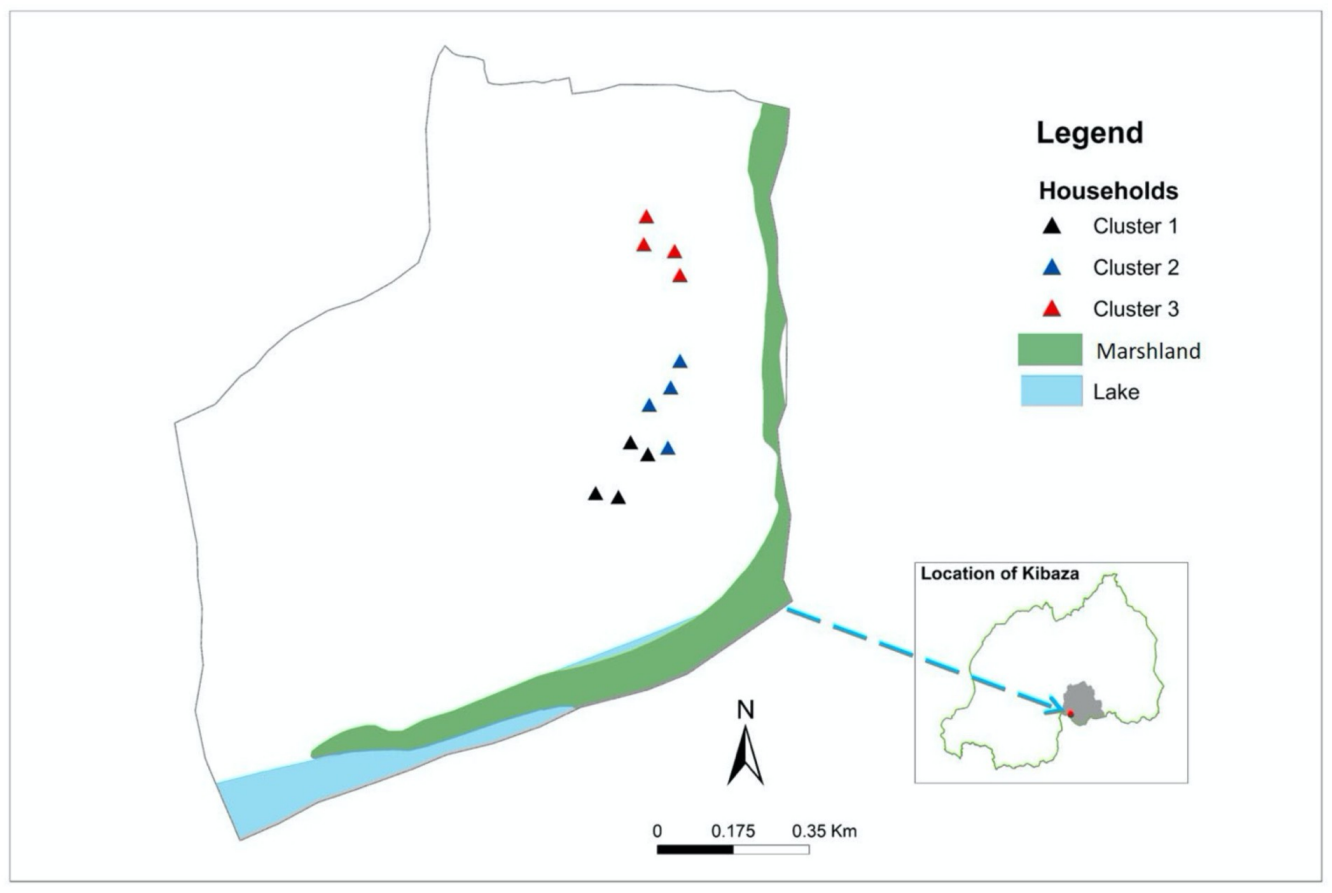
Map of Kibaza showing the three groups of four selected houses (in black, blue and red) included in the study. Inset shows Rwanda in green with Bugesera district in grey and the location of Kibaza village indicated with a red dot.

Field experiments were conducted in Kibaza, Bugesera district, Eastern province, Rwanda. Twelve houses from three village clusters called “isibo” (a cluster of a maximum of 15 households) were selected for mosquito collection (Fig 1). Kibaza village is situated at 1°52’18.0"S and 30°16’11.0"E. The area is a rural, agricultural setting with a traditionally high level of malaria transmission [39]. The study site is characterized by two rainy seasons (March-May and October-December) which alternate with two dry seasons (January-February and June-September). Kibaza is bordered by one irrigated rice scheme with two annual rice growing cycles and the selected houses were mainly made of mud walls with iron sheet roofs.

**Fig 2:**
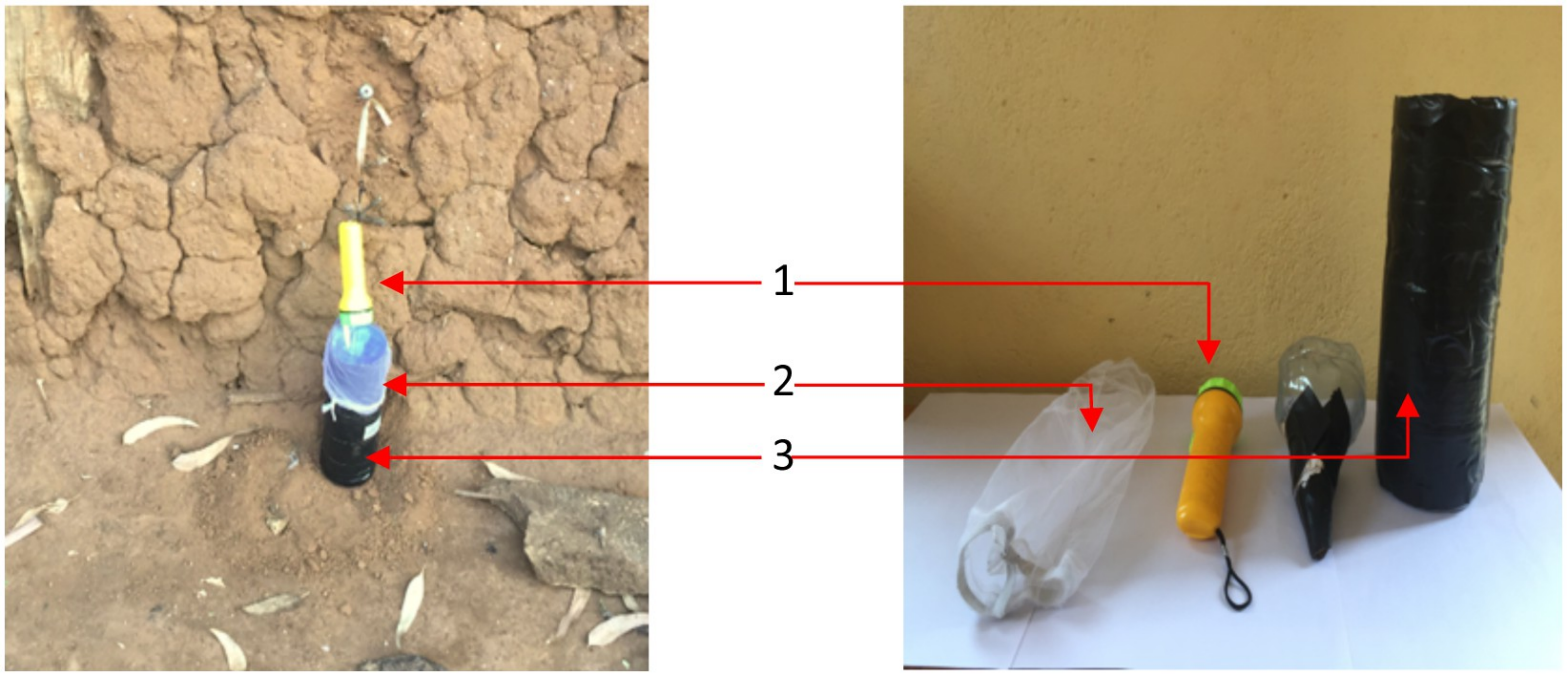
The handmade trap evaluated in the field: (1) torch suspended at 5 centimeters above the trap entrance, (2) gauze net, (3) a ¾ cut plastic bottle wrapped with black scotch tape.

Handmade carbon dioxide-baited mosquito traps were made from 1.5 litre transparent plastic bottles. The top was cut off at three-quarter height and inverted into the remaining part. The opening was elongated with a piece of black paper as a funnel to prevent mosquitoes from escaping from the trap. This design was based on a pilot experiment in the laboratory (see S1 File) in which we evaluated the effectiveness of the handmade trap in terms of collecting female *An*. *coluzzii* in large cages with different sugar sources and heat as stimuli. The trap also included a gauze net that was inserted to prevent the mosquitoes from entering the fermenting solution. In addition, the bottle was wrapped with black scotch tape (Fig 2) [29, 45, 46]. Carbon dioxide was provided by preparing a mixture of 25 g brown sugar, two grams of yeast (Pakmaya instant dry yeast, Istanbul, Turkey) and 250 mL of water [35, 40].

At each house, a handmade carbon dioxide-baited trap was placed either indoors next to a human sleeping under a bed net, or outside of the selected house, preferably near the main entrance of the house and positioned against the wall. The traps were placed on the ground, where the opening of the trap was at 9 cm from the ground (Fig 2). The yeast-sugar mixture was prepared at 9:00 am, and the traps were set up in the bedroom of the community members at the foot end of the bed. At least two occupants slept in the bedroom, protected by an ITN. To examine the effect of light on trap catches, light was provided from a torch (Super bright DH-168 light-emitting diodes (LEDs) powered by 2 X Tiger Head R6S AA UM3 1.5 Volt batteries (last for ± 12 hours). In the experiment with light, the torch was suspended 5 cm above the trap entrance. The torch and the trap were operated from 6:00 pm till 6:00 am.

Three experiments using the bottle trap with either only CO_2_ produced by sugar-yeast fermentation (experiment A), with light alone (experiment B) or with sugar-yeast produced CO_2_ in combination with light (experiment C) were evaluated (Table 1). To control for night and location effects, a 4 x 4 Latin square design was used for comparative studies of mosquito traps at each of the three sites (Table 2). To compare the effectiveness of the three handmade trap designs, CDC light traps were used as a reference. Indoor, CDC light traps were set up in the bedroom and hung at the foot end of the bed, with the shield of the trap at 150 cm from the floor [41]. Outdoor traps were positioned outside the house in the peridomestic area (outside against the wall at the house entrance). First, experiment A was carried out by providing three houses with the yeast-sugar baited trap indoor, three houses with the yeast-sugar baited trap outdoor, three houses with the CDC light trap indoor and three houses with the CDC light trap outdoor. This procedure was repeated for four consecutive nights so that each house had received each of the four trap designs on one night. This procedure was repeated for experiments B and C on different nights. All experiments were carried out between 24 September 2018 and 1 November 2018.

**Table 1:** Experiments carried out during the field phase; N = 12 per experiment.

Experiment	Chemical attractant	Physical attractant
A	Yeast + sugar	-
B	-	Light
C	Yeast + sugar	Light

**Table 2:** Mosquito collection scheme following a 4x4 Latin square design. This schedule was used for the three treatments between 24 September 2018 and 1 November 2018. Experiments were carried out consecutively (not simultaneously).

	*Night 1*	*Night 2*	*Night 3*	*Night 4*
*House 1*, *5 and 9*	Handmade trap indoor	Handmade trap outdoor	CDC light trap indoor	CDC light trap outdoor
*House 2*, *6 and 10*	Handmade trap outdoor	Handmade trap indoor	CDC light trap outdoor	CDC light trap indoor
*House 3*, *7 and 11*	CDC light trap indoor	CDC light trap outdoor	Handmade trap indoor	Handmade trap outdoor
*House 4*, *8 and 12*	CDC light trap outdoor	CDC light trap indoor	Handmade trap outdoor	Handmade trap indoor

The traps were set at 6:00 pm and the owner of the room was instructed to switch off the light of the handmade or CDC trap and tie the bag connected to the collection cup or the net of the handmade traps at 6:00 am the next morning to avoid mosquitoes escaping from the traps. After their collection from the traps, mosquitoes were stored in labelled petri dishes before morphological identification at the laboratory of entomology in Kigali for further analysis.

### Mosquito species identification

Mosquitoes collected per trap per house per night from each experiment were kept separately in labelled petri dishes and were sorted by genus and sex. All female *Anopheles* mosquitoes were morphologically identified to species level using the standard morphological identification keys [42] at the central laboratory. Mosquitoes from each house and trap were then pooled in 1.5 ml labelled vials with silica gel and kept for molecular species identification. A random sample of 250 *An*. *gambiae* s.l. caught indoors and outdoors using CDC light traps and handmade traps were used for DNA extraction and identification using the Polymerase Chain Reaction method (PCR) [43]. DNA was extracted only from the heads and thoraxes of the mosquitoes for amplification. For samples that did not show a product on the electrophoresis gel, legs and wings were tested.

### Statistical analysis

For the field experiments, the numbers of collected female mosquitoes were analysed using different Generalized Linear Mixed Models (GLMM with negative binomial with log function, dispersion estimated) to test the differences in capturing effectiveness of different odour baits in combination with or without visual cue (light) using the handmade trap versus the CDC light traps. The main effects tested were the trap type and the location (indoor/outdoor). Covariates associated with the experimental design (house location, trapping night) were included as random factors in the model. The model, including (significant) covariates, was used to calculate the incidence rate ratios and their 95% confidence intervals. Catches from traps baited with light and with yeast and sugar were not included in the statistical analyses as the number of mosquitoes was too low. A Chi-square test was computed to compare proportions of *An*. *arabiensis* and *An*. *gambiae* s.s. collected with CDC-LT and traps baited with yeast-sugar mixture and light. All statistical analyses were performed using SPSS (Version 25.0, IBM Corporation, New York, USA).

### Ethical approval

The ethical approval was guaranteed to the study (408/CMHS IRB/2016) by the Institutional Review Board of the College of Medicine and Health Sciences, University of Rwanda. Written informed consent was obtained from the head of the selected houses for the experiment.

## Results

### Field experiments

Three mosquito genera were identified from collected mosquitoes (Table 3). Almost two-thirds (n = 2,196, 69.3%) were *Culex* species, followed by *Anopheles* (n = 855, 27%), and *Mansonia* (n = 116, 3.7%). The species collected from the genus *Anopheles* included *An*. *gambiae* sensu lato (n = 742, 86.7%), *An*. *ziemanni* (n = 69, 8%), *An*. *maculipalpis* (n = 39, 4.6%), *An*. *brohieri* (n = 3, 0.4%), *An*. *pharoensis* (n = 1, 0.12%) and *An*. *rufipes* (n = 1, 0.12%).

**Table 3:** Numbers of mosquitoes per genus collected in Kibaza. A, B and C indicate the three different experiments carried out in the field study which each covered 12 trapping nights.

Experiment	Trap type	Location	*Anophele*s spp.	*Culex* spp.	*Mansonia* spp.	Total
A	Sugar-yeast-baited	Indoors	1	1	0	2
	CDC-LT	Indoors	105	65	10	180
	Sugar-Yeast-baited	Outdoors	0	1	0	1
	CDC-LT	Outdoors	124	143	25	292
B	Light-baited	Indoors	0	1	7	8
	CDC-LT	Indoors	67	61	19	147
	Light-baited	Outdoors	1	2	0	3
	CDC-LT	Outdoors	114	401	26	541
C	Sugar-Yeast-Light-baited	Indoors	16	59	3	78
	CDC-LT	Indoors	168	189	8	365
	Sugar-Yeast-Light-baited	Outdoors	22	69	2	93
	CDC-LT	Outdoors	237	1204	16	1457
	TOTAL		855	2196	116	3167
	Species composition (%)		27.0	69.3	3.7	

Of the handmade traps, the trap baited with yeast produced CO_2_ and light (Experiment C) had the highest catch (n = 171) and collected all three genera. This was followed by the light-baited trap (n = 11, experiment B). The trap baited with CO_2_ produced by the yeast-sugar mixture (Experiment A) only collected three mosquitoes over 12 collection nights in total (Table 3).

### Comparison between handmade and CDC light traps

**Fig 3:**
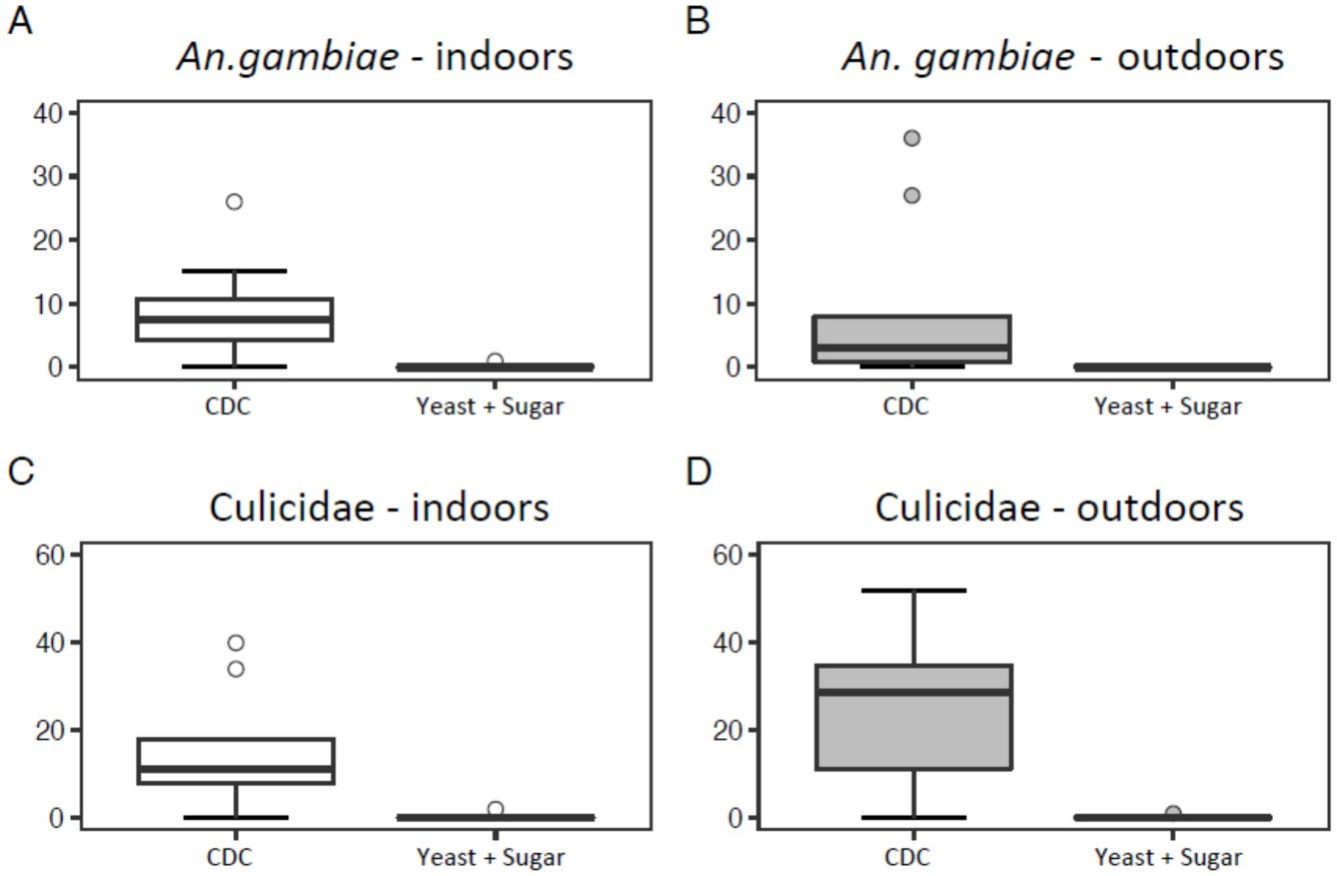
Boxplots showing the number of *Anopheles gambiae* s.l. (A and B) and Culicidae (C and D) collected using a CDC light trap (12 trapping nights per treatment) or a trap baited with a sugar and yeast mixture (12 trapping nights per treatments) in Rwanda.

**Fig 4:**
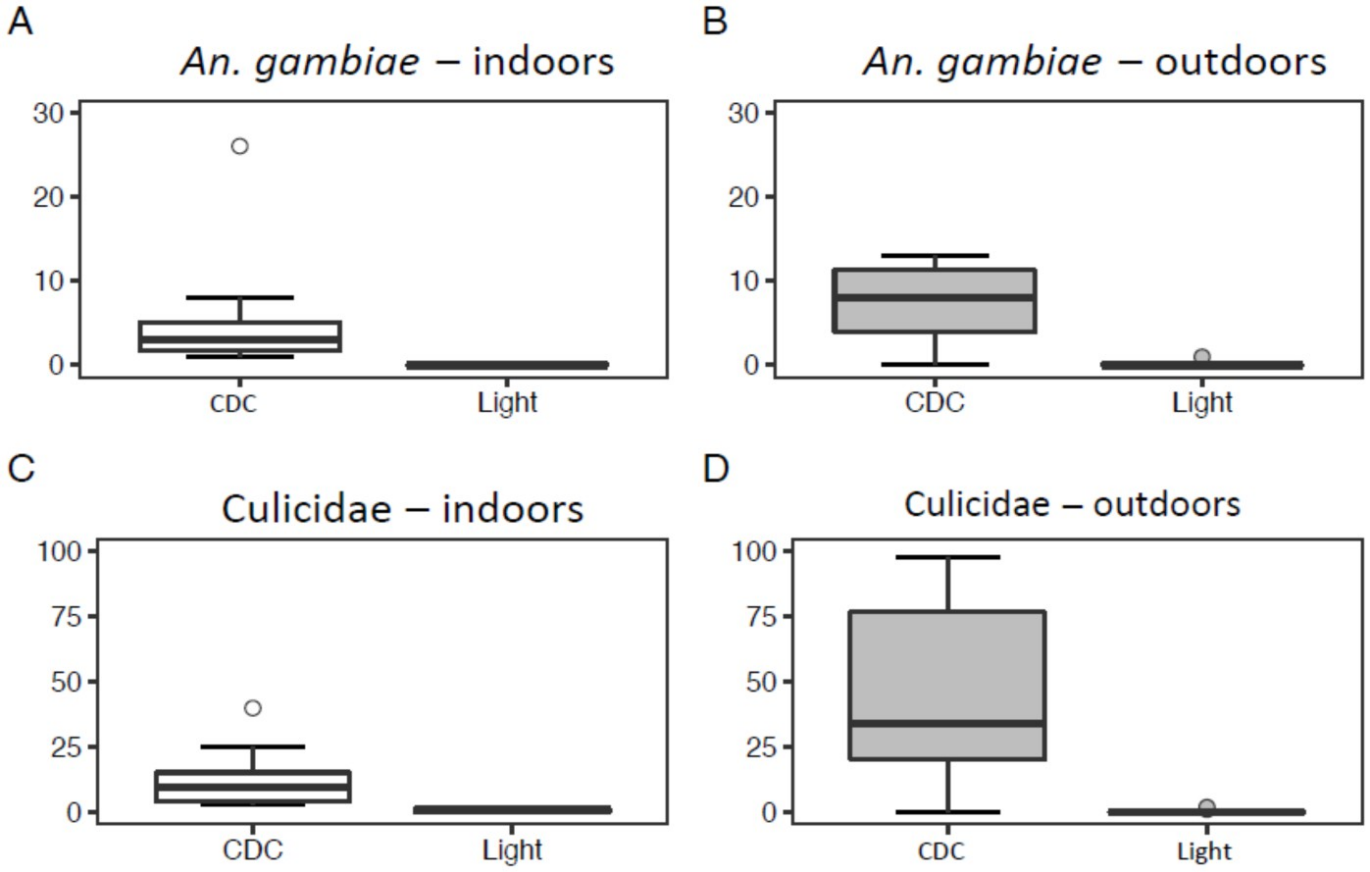
Boxplots showing the number of *Anopheles gambiae* s.l. (A and B) and Culicidae (C and D) collected using a CDC light trap (12 trapping nights per treatment) or a trap baited with light (12 trapping nights per treatments) in Rwanda.

**Fig 5:**
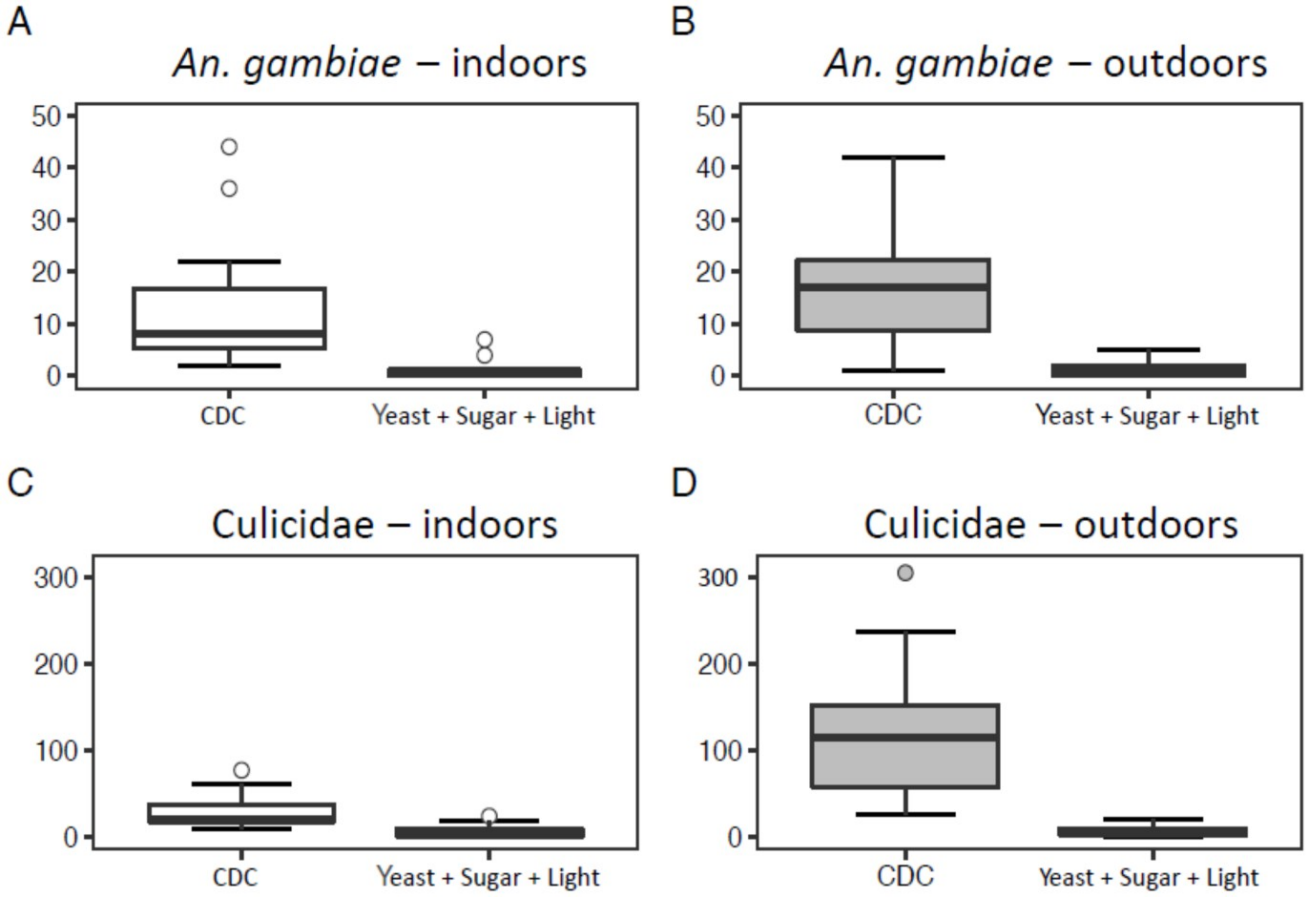
Boxplots showing the number of *Anopheles gambiae* s.l. (A and B) and Culicidae (C and D) collected using a CDC light trap (12 trapping nights per treatment) or a trap baited with the combination of a sugar-yeast mixture and light (12 trapping nights per treatments) in Rwanda.

CDC light traps collected significantly higher numbers of female mosquitoes (Culicidae) and *An*. *gambiae* s.l. (GLMM, *P* < 0.001; Table 4) compared to all three handmade traps baited with sugar and yeast alone (Fig 3), light alone (Fig 4), or with the combination of sugar, yeast and light (Fig 5).

**Table 4:** Parameter estimates for the effects of trap type and location on numbers of mosquitoes (Culicidae and *An*. *gambiae* s.l.).

Experiment	Mosquito group	Trap type and Location	Beta	Exp(B)	95% CI	*P*
**A**	Culicidae	Sugar+Yeast	-5.107	0.006	0.002–0.020	< 0.001
		CDC-LT	*			
		Indoors	-0.361	0.697	0.340–1.429	0.325
		Outdoors	*			
	*An*. *gambiae* s.l.	Sugar+Yeast	-5.486	0.004	0.0005–0.035	< 0.001
		CDC-LT	*			
		Indoors	0.303	1.354	0.559–3.280	0.502
		Outdoors	*			
**B**	Culicidae	Light	-3.964	0.019	0.008–0.044	< 0.001
		CDC-LT	*			
		Indoors	-0.729	0.483	0.233–1.001	0.050
		Outdoors	*			
	*An*. *gambiae* s.l.	Light	-5.008	0.007	0.001–0.033	< 0.001
		CDC-LT	*			
		Indoors	-0.409	0.665	0.338–1.307	0.236
		Outdoors	*			
**C**	Culicidae	Sugar+Yeast+Light	-2.135	0.118	0.066–0.213	< 0.001
		CDC-LT	*			
		Indoors	-0.836	0.434	0.241–0.779	0.005
		Outdoors	*			
	*An*. *gambiae* s.l.	Sugar+Yeast+Light	-2.357	0.095	0.051–0.176	< 0.001
		CDC-LT	*			
		Indoors	-0.137	0.872	0.479–1.587	0.653
		Outdoors	*			

The number of mosquitoes (Culicidae) caught indoors was significantly lower than those collected outdoors for the trials in which we tested light only (GLMM, *P* = 0.050), or the combination of sugar, yeast and light (GLMM, *P* = 0.005). This was not the case for numbers of *An*. *gambiae* s.l. or for the experiment with sugar and yeast only (Table 4).

All models reported in Table 4 included the random factors house location and trapping night (collection date). In experiments B and C, these random factors were not significant, but for experiment A, collection date was significant in the model for all mosquitoes (GLMM, Wald chi-square = 4.320, *P* = 0.038), while house location was significant in the model for *An*. *gambiae* s.l. (GLMM, Wald chi-square = 4.477, *P* = 0.034).

**Fig 6:**
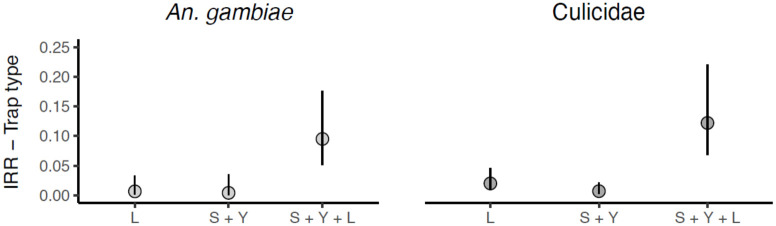
Estimated incidence rate ratio’s (IRR, Table 4) and their 95% confidence intervals for the main effect of trap type (handmade trap versus CDC light trap) for the numbers of female *An*. *gambiae* (left panel) and Culicidae (right panel). L: handmade trap baited with light only, S + Y: handmade trap baited with a sugar-yeast mixture, S + Y + L: handmade trap baited with a sugar-yeast mixture and light.

When comparing the overlap in 95% confidence intervals of the incidence rate ratio’s, we can deduce that traps baited with the combination of sugar-yeast mixture and light were significantly better in catching mosquitoes (Culicidae) as well as in catching *An*. *gambiae* s.l. than traps baited with a sugar-yeast mixture only, or light only (Fig 6).

### Sibling species identification

Of a random sample of 250 female *An*. *gambiae* s.l. identified to sibling species by PCR, 4% (11/250) were *An*. *gambiae s*.*s*. and 96% (239/250) were *An*. *arabiensis*. There was no significant difference between the relative proportions of both species collected in CDC traps and in traps baited with a sugar-yeast mixture and light (Chi-square = 0.206, *P* = 0.650). Similarly, the relative proportion of both sibling species in indoor and outdoor collections was the same (Chi-square = 2.134, *P* = 0.144).

## Discussion

Many studies have demonstrated that sugar-fermenting yeast is a practical source of CO_2_ in traps, especially in the field, because industrial CO_2_ is not always available and is costly [35, 40, 44]. However, its potential for use in simple, handmade traps for mosquito surveillance in a citizen science context had not been fully explored. Overall, traps baited with a yeast-sugar mixture and light, a yeast-sugar mixture alone, or with light alone caught very few mosquitoes compared to (powered) CDC light traps. Although this study investigated the effects of CO_2_ produced by the fermentation of brown sugar and dry yeast in capturing adult mosquitoes, cues such as light added to the trap seemed to play a role in capturing mosquitoes. When comparing the three handmade traps among each other, the trap baited with a yeast-sugar mixture and light captured more *An*. *gambiae* s.l. The same was true for capturing other Culicidae, which included species such as *Cx*. *quinquefasciatus*, *Cx*. *annulioris*, *Mansonia africana* and *M*. *uniformis*. Thus, malaria vectors and other mosquitoes seem to make use of visual cues in host-seeking despite their nocturnal habit [29], but here only in combination with CO_2_. Interestingly, traps baited with a yeast-sugar mixture and light caught significantly more mosquitoes outdoors than indoors, which agrees with previous research [45]. However, traps baited with a yeast-sugar mixture did not catch more mosquitoes outdoors and these findings therefore disagree with other studies conducted previously [45, 46].

It is clear that CDC traps caught the highest number of mosquitoes, as well as a higher diversity of species. It should be noted that the CDC trap requires a powered fan, whereas the handmade trap is a non-mechanical, passive trap without an active suction mechanism. Interestingly, both indoors and outdoors, the number of *An*. *gambiae* s.l. in the handmade trap with light and CO_2_ was about 9–10% of the number caught with a CDC light trap (16 out of 168 indoor, and 22 out of 237 outdoor; experiment C). This suggests that 10 bottle traps distributed over 10 houses would capture a similar number of mosquitoes (including *An*. *gambiae*) as one CDC light trap placed in one house. In other words, if sufficient volunteers can be recruited in a citizen science project using a bottle trap, this could present an alternative for installment of a CDC light trap. Further studies are needed to compare the cost-effectiveness of both approaches, and should investigate labour, logistical costs and materials, especially because plastic bottles will be banned in the country. Alternative and more sustainable materials, such as bioplastics, would thus need to be identified. In addition, longevity of the batteries and the torch, and inclusion of human foot odor collected on socks as an attractant are issues or options to further explore to improve the durability and capture efficiency of the traps.

If such citizen science reporting can be linked with digital technology (e.g. reporting observations through a mobile app), rapid assessments of malaria risk can be made with high spatial coverage. In that respect, our recent work presents the results of a one-year study in which 116 volunteers participated in the collection of mosquito data using the above-described handmade traps [47]. It demonstrated significant correlations between numbers of Culicidae caught in the passive trap and confirmed malaria cases. The study concluded that a citizen science approach can contribute to mosquito monitoring, and can help to identify areas that, in view of limited resources for control, are at higher risk of malaria.

Traps can be further developed by evaluating the effectiveness of the handmade trap for *Anopheles* mosquitoes in other malaria endemic areas in Rwanda, as well as in other regions with different vector species and different seasonality. Such evaluations need to include concerns about data quality, standardization, as well as the earlier mentioned need to replace components of the trap.

## Conclusion

A handmade trap that produced CO_2_ by yeast-sugar fermentation attracted and trapped mosquitoes, including malaria vectors, in the field in Rwanda. Additional visual cues such as a light source increased the attractiveness of the trap for mosquitoes. Although there are limitations with using the handmade trap, the trap presents an alternative option for inclusion in mosquito surveillance activities in a citizen science context in rural areas.

## Supporting information

S1 File Results of pilot experiment in the laboratory. The experiment evaluated the effectiveness of the handmade trap in terms of collecting female *An*. *coluzzii* in large cages with different sugar sources and heat as stimuli.
(DOCX)
