# Supplementary material for: A handmade trap for malaria mosquito surveillance by citizens in Rwanda
Source: PLoS One. 2022 May 11;17(5):e0266714. doi: 10.1371/journal.pone.0266714 (PMC9094558; doi:10.1371/journal.pone.0266714)
Supplement: S1 File — The experiment evaluated the effectiveness of the handmade trap in terms of collecting female An. coluzzii in large cages with different sugar sources and heat as stimuli. (DOCX) [file pone.0266714.s001.docx]

**Additional File 1**

**Laboratory experiments**

A series of pilot experiments was conducted at the Laboratory of Entomology of Wageningen University & Research, the Netherlands. A handmade carbon dioxide-baited mosquito trap was made from a 1.5 litre transparent plastic bottle. The top was cut off at three-quarter height and inverted into the remaining part. The opening was elongated with a piece of black paper as a funnel to prevent mosquitoes from escaping from the trap. One hundred non-fed female *An. coluzzii* were obtained as described by Smallegange et al. (2010) and placed in small release cages. The age of the female mosquitoes used was 8-10 days. Mosquitoes were hydrated with water-moisturized cotton wool on top of the release cage 16 h before the experiments were started.

Experiments were carried out to determine the attractiveness of traps baited with CO_2_ produced by (1) yeast and sugar, (2) yeast, sugar and heat, or (3) yeast and molasses (Table 1). For experiments (1) and (2) the CO_2_ was obtained by mixing 6 g of dry yeast (Dr. Oetker, The Netherlands), 25 g of brown sugar (Van Gilse Kristalsuiker, Suiker Unie, The Netherlands) and 250 ml of tap water. For experiment (2), a heat cable was wrapped around the bottle and set at 37 °C to mimic human body temperature to attract the mosquitoes to the trap. For experiment (3), 4.4 g of dry yeast (Dr. Oetker, The Netherlands) were mixed with 62.5 g of molasses (Golden Molasses Unsulfured, Sweet Harvested, Natural American Foods^™^) and 500 ml of tap water [39]. Mixing of sugar or molasses with water and yeast took place 1-1.5 h before mosquitoes were released. No additional stirring was done during the experiments. A Xentra 4100 CO_2_ analyser (Servomex, the Netherlands) was used to check the production of carbon dioxide in each experiment. After measuring the flow rate of CO_2_, the trap was placed at the center of a textile screen cage (330 X 250 X 233 cm; Howitec Netting BV, The Netherlands) which was situated inside a larger climate-controlled room (22.2 ± 1.6 °C and 52.6 ± 7.8% RH). The experiments were conducted in the last four hours of the dark phase when *An. coluzzii* is normally searching for a host [40–42]. The experiments were alternated to rule out day effects. Each experiment was replicated three times (see Table 1) except for molasses the treatment was replicated twice. For each replicate, the 100 mosquitoes were released from their release cage placed in the right corner of the screen cage and left overnight (24 h). Afterwards, mosquitoes remaining in the release cage and in the trap were counted.

**Table 1.** Use of attractants in each of the experiments conducted in the laboratory phase

| **Experiment** | **Replicates** | **Chemical attractant** | **Physical attractant** |
| --- | --- | --- | --- |
| **1** | 3 | Yeast + sugar |  |
| **2** | 3 | Yeast + sugar | Heat (37 ^0^C) |
| **3** | 2 | Yeast + molasses |  |

**Results**

Of the 778 *An. coluzzii* released for all the screen-cage experiments, 123 (15.8%) *An. coluzzii* were re-captured over a 24-hr period. The mean trap entry response for a trap baited with sugar and yeast was 0.18 ± 0.057, 0.06 ± 0.026 for a trap baited with yeast, sugar and heat, and 0.19 ± 0.053 for a trap baited with molasses and yeast. There was a significant difference among treatments on catches of *An. coluzzii* (GLM; df = 2; *P* = 0.028). The effect of CO_2_ release was included as a covariate in the final model, and although not significant (GLM; df = 1; *P* = 0.192), this gave the model the lowest AIC value and best fit. Our LSD post-hoc test showed that the trap baited with molasses and yeast caught significantly higher proportions of *An. coluzzii* than the trap baited with yeast, sugar and heat (*P* = 0.015). All other comparisons were not significant. For practical purposes (sugar being more readily available than molasses), our choice favoured the yeast and sugar mixture for carbon dioxide production above the other two treatments for further evaluations in the field. Heat was not included as a potential attractive cue in the field experiments.
